# A comparative study of the essential oil extracts of *Centaurea alexanderina* different parts: GC-MS analysis, antimicrobial, antioxidant, and anti-hyperglycemic activities

**DOI:** 10.1186/s12906-025-04805-1

**Published:** 2025-03-05

**Authors:** Hossam A. Abdallah, Naglaa Afifi, Enas I. A. Mohamed, Mohamed Sebak, Rabab Mohammed, Mohamed A. Zaki

**Affiliations:** 1 Department of Pharmacognosy, Faculty of Pharmacy, Beni-Suef University, Beni-Suef, 62514 Egypt; 2 Microbiology and Immunology Department, Faculty of Pharmacy, Beni-Suef University, Beni-Suef, 62514 Egypt

**Keywords:** *Centaurea alexanderina*, Essential oil extract, Antioxidant, Antimicrobial, Anti-hyperglycemic, Docking

## Abstract

**Background:**

Natural products have been a cornerstone in the field of drug discovery for centuries, they have provided numerous therapeutic agents that have significantly impacted modern medicine. *Centaurea alexanderina* is a canescent herb that is native to Egypt and Libya and reported in Egyptian folk medicine as an anti-hyperglycemic, antioxidant, and antimicrobial herb. It is an understudied species, thusly, the target of this contribution was to perform a comparative study of the influence of plant part variation on its chemical and pharmacological characteristics. In this context, essential oil extracts from different parts of *C. alexanderina* (flowers, stems, leaves, and roots) have been analyzed chemically and tested for their antioxidant, antimicrobial, and anti-hyperglycemic activities.

**Methods:**

The essential oil extracts from different parts of *C. alexanderina* have been prepared and their chemical compositions have been analyzed using GC–MS technique. Antimicrobial activities of different essential oil extracts were evaluated via the agar cup diffusion method. DPPH radical scavenging and FRAP assays were used for determination of the antioxidant capacity. Anti-hyperglycemic activities of the four essential oil extracts under study were investigated via *α*-glucosidase inhibition assay. A computational study has been performed using molecular docking of the identified compounds in the most active essential oil extracts (leaves and roots) against *α*-glucosidase enzyme using PDB entry 5NN8.

**Results:**

A GC–MS analysis of the four essential oil extracts revealed high levels of non-terpenoid hydrocarbons in three essential oil extracts (flower, stems, and leaves) and alcohols in roots essential oil extract, followed by fatty acids in flowers, diterpenoids in stems, steroids in leaves, and fatty acid esters in roots. Roots and stems essential oil extracts exhibited selective activity against *Pseudomonas aeruginosa* (*P. aeruginosa*), whereas leaves essential oil extract showed activity against both *Salmonella enterica* (*S. enterica*) and *Escherichia coli* (*E. coli*). Essential oil extracts of different parts of the plant exhibited DPPH free radical scavenging activity with IC_50_ values of 128, 134, 152, and 163 μM for flowers, roots, stems, and leaves essential oil extracts; respectively, while in FRAP assay, the results showed different pattern; flowers revealed the highest activity followed by leaves, roots, and stems (107.50 ± 3.64, 125.80 ± 4.2, 139.4 ± 5.27, and 155.59 ± 5.27 ug/mL, respectively). In vitro evaluation of anti-hyperglycemic activity via inhibition of *α*-glucosidase enzyme assay unveiled leaves and roots essential oil extracts as the most inhibitors with IC_50_ values of 349 and 369 μg/mL; respectively. The molecular docking study of detected metabolites in the most active essential oil extracts against human *α*-glucosidase enzyme (PDB: 5NN8) revealed moderate to high binding affinities (-4.6 to -7.3 kcal/mol for leaves and -4.4 to -7.9 kcal/mol for roots essential oil metabolites).

**Conclusion:**

Current research demonstrated the variations in chemical composition and biological activities of essential oil extracts from different parts of *Centaurea alexanderina* and reported anti-hyperglycemic, antioxidant, and antimicrobial potentials of the different essential oil extracts.

**Supplementary Information:**

The online version contains supplementary material available at 10.1186/s12906-025-04805-1.

## Introduction

The Asteraceae family is highly significant as a source of active secondary metabolites, containing plants with diverse biological activities. *Centaurae* is one of the largest genera within the family comprising more than 500 species worldwide particularly across the Mediterranean region and Western Asia [1–3]. Several *Centaurae* species are well known for their traditional use in the treatment of various diseases [4]. Members of this genus contain a variety of secondary metabolites including sesquiterpene lactones, flavonoids, lignans, alkaloids, simple phenolics, steroids, triterpenes, hydrocarbons, polyacetylenes, and anthocyanins. Secondary metabolites obtained from *Centaurea* species have been the focus of research due to their biological activities; antibacterial, antifungal, anti-plasmodial, cytogenetic, antiulcerogenic, and antioxidant activities. They also have cytotoxic, antiviral, antiprotozoal, anti-inflammatory, and antidiabetic activities [2, 5]. *Centaurea alexanderina* Delile is a canescent biennial herb, 15–40 cm tall, it is characterized by a thick fleshy rootstock, branched stems, sulcate, pinnatifid to pinnatisect, dentate, mucronate, and petiolate leaves. Capitula are single or paired at the tips of the branches with dirty yellow corolla and purple-brown anthers [6]. It is native to Egypt and Libya as recorded from Mediterranean phytogeographical region [7]. *C. alexandrina* has been used as anti-hyperglycemic in Egyptian folk medicine [8]. Previous reports indicated hypoglycemic, anti-inflammatory, anticancer, and analgesic activities and several bioactive compounds from the species including flavonoids, sesquiterpenoids, and lignans [9].

The essential oil extract, often referred to as “absolute” is the most concentrated mode of natural aroma, it is most frequently utilized as a plant scent isolate in food and aroma industries [10]. The most popular technique for producing the essential oil extract is the use of organic solvents then extracting with absolute ethanol to eliminate waxy components and obtain the essential oil extract, which is the accurate reflection of a plant's aroma [10]. Since the essential oil yield of *Centaurea* species is low and essential oil extract preparation techniques are inexpensive, easy-to-use, and readily available [10, 11], authors pursued the current work on *C. alexanderina* essential oil extracts.

Notably, *C. alexandrina* is yet an understudied species. Previous work on the plant was focused mainly on aerial parts [9, 11, 12]. To the best of our knowledge, this is the first report of essential oil extracts of different parts of *C. alexandrina.* The target of this contribution was to perform a comparative study of the influence of plant part variation on the characteristics of its essential oil extracts, focusing not only on qualitative and quantitative investigation of chemical constituents of the essential oil extracts obtained from different parts of *C. alexandrina* but also on their biological activities.

*C. alexandrina* has been traditionally used in Egyptian folk medicine for its anti-hyperglycemic properties [8]. Given this, we focused on investigating its anti-hyperglycemic activity to identify which plant parts or specific metabolites are responsible for this effect. Maintaining normal blood glucose levels is crucial because elevated glucose levels can lead to a reduction in plasma antioxidant levels. Antioxidants play a key role in preventing the progression of diabetes by neutralizing oxidative stress [13]. Additionally, there is a bidirectional relationship between diabetes and microbial infections. Diabetic patients, being immunocompromised and chronic in nature, are more susceptible to microbial infections. Therefore, exploring the antimicrobial activities of approved antidiabetic agents can be highly valuable for clinicians. The preferential use of antidiabetic agents with dual action, both anti-hyperglycemic and antimicrobial, could offer multiple benefits for diabetic patients, such as enhanced protection against infections and reduced overall treatment costs [14].

## Materials and Methods

### Chemicals and reagents

All solvents used in the study were of high analytical grade. Ethanol, *n*-hexane, methanol, FeCl_3_.6H_2_O, NaOH, and HCl were purchased from El-Nasr company for pharmaceuticals and chemicals (Egypt). DMSO, 2,4,6-tripyridyl s-triazine (TPTZ), *p*-nitrophenyl-*α*-D-glucopyranoside, acarbose, and ascorbic acid were purchased from Merck (Rahway, NJ, USA) and Sigma-Aldrich (St. Louis, MO, USA).

### Plant material

In Summer of 2022, *C. alexanderina* plant was collected from North coast of Egypt (N 28° 2′ 55.7376", E 34° 26′ 13.7328"). The collection of *C. alexanderina* plant and experimental work complied with relevant institutional, national, and international guidelines and legislation. Taxonomical identification of the plant material was done by Prof. Dr. Abdalhalim Mohamed (Flora & Phyto-taxonomy Researches, Horticulture Research Institute, Agricultural Research Center, Giza, Egypt). Voucher specimen (BUPD-89) was deposited in Pharmacognosy Department, Faculty of Pharmacy, Beni-Suef University, Beni-Suef, Egypt.

### Essential oil extract preparation

Fifty grams from the fresh flowers, stems, and leaves and twenty-five grams from roots were subjected separately to solvent extraction starting with macerating the fresh plant parts in hexane solvent at room temperature. The yield obtained after filtration was re-extracted with absolute ethyl alcohol, followed by drying to afford essential oil extracts weighing (0.34, 0.25, 0.47, and 0.17 mg; respectively). Each essential oil extract was preserved in a sealed dark glass vial at 4 °C till required [15, 16].

### Gas chromatography–Mass spectrometry analysis

Investigation of the chemical composition of the four essential oil extracts under study was performed using GC–MS system: thermo scientific trace 1310 Gas Chromatograph coupled with ISQ LT single quadrupole mass spectrometer. Column: db5-ms, 30 m; 0.25 mm id (J&W scientific). Ionization mode: EI. Ionization, voltage: 70 eV. Temperature program: 40 ºC (3 min.)—280 ºC (5 min.) at 5 ºC/min.—290 ºC (1 min.) at 7.5 ºC/min. Detector temperature: 300 ºC. Injector temperature: 200 ºC. Carrier gas: helium; flow rate 1 mL/min. 10 µg sample dissolved in 1 mL methanol; injection volume was 1 µL. The structures of identified compounds were proposed on the basis of comparing individual peaks with Wiley & NIST computer mass libraries [17].

### Screening of antimicrobial activity and determination of MIC

The antimicrobial potential of various essential oil extracts was investigated using the agar diffusion assay with minimal changes [18]. The antibacterial activity of different essential oil extracts was assessed against* Listeria monocytogenes* (ATCC 7644), *Enterococcus faecalis* (V583), *Salmonella enterica* (ATCC 14028), *Escherichia coli* (ATCC 25922), and *Pseudomonas aeruginosa* (ATCC 27853), whereas their antifungal activity was evaluated using *Candida albicans* (ATCC 60193). Briefly, the tested microbial strains were suspended in saline until they reached 0.5 McFarland turbidity, and then they were streaked using a sterile cotton swab on the surface of Mueller–Hinton agar (MHA), followed by creating 10-mm wells in the agar plates using a sterile borer. Subsequently, the pre-inoculated microbial strains were treated with 100 µL of each essential oil extract at a concentration of 1 mg/mL in dimethyl sulfoxide (DMSO). The inoculated MHA plates were then left for 100 min. at 4 °C before being incubated overnight at a temperature appropriate for microbial growth. After the overnight incubation, the zones of inhibition were measured, and the antimicrobial potentialities of the four essential oil extracts were recorded accordingly. Additionally, DMSO was employed as a negative control, while standard antibacterial and antifungal agents included ciprofloxacin and nystatin, respectively.

Next, the bioactive parts at a concentration of 1 mg/mL were subsequently examined for their minimum inhibitory concentration (MIC) against the corresponding sensitive microbial strains using the agar dilution assay [19]. The MIC values of leaves and stems essential oil extracts were measured after serial dilution of their active concentration (1 mg/mL) from 500 µg/mL to 62.5 µg/mL, whereas the roots essential oil extract MIC value was assessed using serial dilutions from 125 µg/mL to 32.25 µg/mL because of its low dry weight supply. Firstly, the different samples were prepared at stock concentration of 20 mg/mL in DMSO. Then, the molten MHA was seeded with specific volumes from the stock solutions to reach the final desired concentrations described above. Afterwards, the sensitive bacterial strains (1.5 × 10^6^ CFU/mL) were administered to the surface of the MHA plates pre-seeded with the serial dilutions of the essential oil extract using a sterile metal multipoint inoculator [20]. At last, the inoculated MHA plates were overnight incubated, and the MIC values of the active samples were calculated as the lowest concentration of essential oil extracts that caused complete inhibition of microbial growth.

### Antioxidant activity

#### DPPH radical-scavenging activity

Somanjana Khatua et al. approach [21] was used to determine the effectiveness in the 2,2'-diphenyl-1-picrylhydrazyl (DPPH) radical scavenging assays for the essential oil extracts of different parts of *C. alexanderina*. In brief, 300 μL of each sample were mixed with 3.0 mL of 250 μM DPPH in absolute ethanol and the mixture was shaken and allowed to stand for 30 min. in the dark at room temperature, and finally, absorbance was recorded at 595 nm wavelength. The degree of scavenging was calculated by Equation No.1

1 $$\text{Scavenging effect }(\%)=\frac{(\text{Absorbance of control}-\text{ Absorbance of sample})}{\text{Absorbance of control }}\times 100$$

[Where “Absorbance control” is the absorbance of the control reaction comprising all reagents but excluding the test sample, and “Absorbance of sample” is the absorbance of the tested essential oil extract]. IC_50_ values were calculated from the dose–response curve via interpolation from the linear regression analysis.

#### Ferric Reducing Antioxidant Power (FRAP) Assay

FRAP assay was used to determine the ferric reducing activity of the different essential oil extract samples. it was carried out in accordance with Hidalgo, G.-I., and Almajano [22] with minor modification. 300 mM acetate buffer (pH 3.6) with 10 mM 2,4,6-tripyridyl s-triazine (TPTZ) solution, and 20 mM FeCl_3_.6H_2_O were mixed (10:1:1; respectively) and heated to 37 ℃ in a water bath to form FRAP reagent. For the analysis, 10 µL of essential oil extract samples (1 mg/mL, dissolved in methanol: ultrapure water: 1M HCl; 70: 29.5: 0.5) were added to 190 μL of FRAP reagent and then incubated at 37 °C temperature for 30 min. The absorbance of reaction mixture was measured spectrophotometrically at 594 nm. The analysis was performed in triplicate using ascorbic acid as a standard.

### *α*-Glucosidase inhibitory assay

*α*-Glucosidase inhibitory assay was carried out according to reported method with minor modifications [23]. In a 96-well plate, reaction mixture containing 60 μL *α*-glucosidase (K938-100, BioVision®, Inc.) in a phosphate buffer (0.3 U/mL), and 10 μL of varying concentrations of test agent (5 µg/mL in methanol) was preincubated at 37 °C for 15 min. Then, 150 μL of *p*-nitrophenyl-*α*-D-glucopyranoside (P-NPG) (1 mM) was added as a substrate and incubated further at 37 °C for 40 min. The reaction was stopped by adding 150 μL NaOH (50 mM). The absorbance of the released *p*-nitrophenol was measured at 405 nm using ELISA Reader. Acarbose at various concentrations (0.1– 0.5 mg/mL) was included as a standard. The enzyme together with P-NPG was set up in parallel as a control and each experiment was performed in triplicates. The results were expressed as percentage inhibition, which was calculated using Equation no.2.

2 $$\text{Inhibitory activity }(\%)=\left[1-\left(\frac{As}{Ac}\right)\times100\right]$$

where, As is the absorbance in the presence of test substance and Ac is the absorbance of control.

### Molecular docking studies

Identified compounds in the most active essential oil extracts (leaves and roots) of *C. alexanderina* following GC–MS analysis were docked into *α*-glucosidase enzyme sites (active site and secondary substrate-binding site) using PDB entry 5NN8 [24] that was retrieved from Protein Data Bank (https://www.rcsb.org/). PDB entry 5NN8 has been selected according to certain required parameters such as source of the enzyme (human), resolution (around 2 Å or less), and to be recently uploaded. Structures of tested compounds were downloaded from PubChem [25] [August, 2023] followed by minimization of their energies using Chem Bio 3D (Chem Bio Office Ultra 12.0 suite). Docking studies were accomplished using Autodock Vina in Pyrx (version 0.8), “https://pyrx.sourceforge.io” [26]. XYZ coordinates were set as; -14.255, -28.033, 94.787 for active site and 4.042, -57.627, 70.382 for secondary substrate-binding site. BIOVIA Discovery Studio visualizer v21.1.0.20298 (Dassault systems Biovia Corp., San Diego, CA, USA) and Pymol software [27] were used for visualization and analysis of the docked ligands poses.

### Statistical analysis

All results of the performed assays were expressed as mean ± standard deviation (SD) from three separate experiments. IC_50_ values were calculated from dose response curve utilizing non-linear regression analysis.

## Results

### Chemical composition of essential oil extracts from different parts of *C. alexanderina* identified by GC–MS

**Fig. 1:**
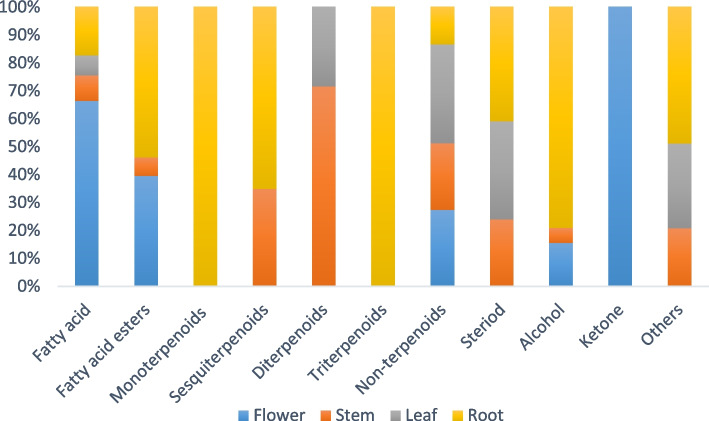
Relative percentage of classes of detected compounds in essential oil extracts from different parts of *C. alexanderina* by GC–MS

To compare essential oil extracts from different parts of *C. alexanderina* in terms of phytochemistry, we sampled essential oil extracts produced from freshly harvested *C. alexanderina* flowers, stems, leaves, and roots. The yield of prepared essential oil extracts was found to be 0.69, 0.5, 0.93, and 0.68% of the different fresh parts; respectively. GC–MS was used to determine the chemical composition of the four essential oil extracts (Fig. 1S). A marked difference in the GC chemical profile of the different essential oil extracts could be noticed from Table 1 and Fig. 1; the identification of the compounds was based on comparison of revealed data with Wiley & NIST computer mass libraries; this included retention time, percentage of peak area, molecular weight, and molecular formula.

**Table 1:** Chemical composition of essential oil extracts from different parts of *C. alexanderina* (identified by GC–MS)

NO	Compound	M.F	M.WT	R.T (min)	Part used (Area %)			
					**Flowers**	**Stems**	**Leaves**	**Roots**
A. Fatty acid								
1	*Cis*-5, 8, 11, 14, 17-eicosapentaenoic acid	C_20_H_30_O_2_	302	4.76	-	-	-	0.23
2	Erucic acid	C_22_H_42_O_2_	338	23.59	0.89	-	-	-
3	Hexadecanoic acid	C_16_H_32_O_2_	256	27.16	10.86	-	-	-
4	Oleic Acid	C_18_H_34_O_2_	282	27.60	0.99	-	-	3.58
5	*Cis*-11-eicosenoic acid	C_20_H_38_O_2_	310	27.63	-	1.98	-	-
6	*Cis*-13-eicosenoic acid	C_20_H_38_O_2_	310	27.63	-	-	1.57	-
7	9,12-Octadecadienoic acid (*Z*,*Z*)	C_18_H_32_O_2_	280	30.85	1.69	-	-	-
					**14.43**	**1.98**	**1.57**	**3.81**
B. Fatty acid esters								
8	2,3-Dihydroxypropyl ester, (*Z*,* Z*, *Z*)—9, 12, 15-octadecatrienoic acid	C_21_H_36_O_4_	325	4.02	-	-	-	0.45
9	6,9,12,15-Docosatetraenoic acid methyl ester	C_23_H_38_O_2_	346	5.13	-	-	-	0.32
10	10-Heptadecen-8-ynoic acid methyl ester (*E*)	C_18_H_30_O_2_	278	5.39	-	-	-	0.38
11	5,8,11,14-Eicosatetraenoic acid, methyl ester.(all-*Z*)	C_21_H_34_O_2_	318	5.45	-	-	-	0.19
12	*Cis*-2-phenyl-1, 3-dioxolane-4-methyl octadec-9, 12, 15-trienoate	C_28_H_40_O_4_	440	5.51	-	-	-	0.29
13	9-Octadecen-12-ynoic acid, methyl ester	C_19_H_32_O_2_	292	6.96	-	-	-	0.33
14	Methyl 16-hydroxy-hexadecanoate	C_17_H_34_O_3_	286	26.33	-	-	-	2.3
15	Hexadecadienoic acid, methyl ester	C_17_H_30_O_2_	266	29.47	-	-	-	0.55
16	Cyclopropanedodecanoic acid, 2-octyl-, methyl ester	C_24_H_46_O_2_	312	30.11	-	-	-	1.13
17	Ethyl (9*Z*,12*Z*)-9,12-octadecadienoate	C_20_H_36_O_2_	308	30.37	9.79	-	-	-
18	2-[[2-[(2-ethylcyclopropyl)methyl) cyclopropyl] methyl]-, methyl ester cyclopropaneoctanoic acid	C_22_H_38_O_2_	334	30.61	0.46	-	-	-
19	Hexadecanoic acid, 2,3-dihydroxypropyl ester	C_19_H_38_O_4_	330	32.28	-	-	-	0.58
20	Docosanoic acid, 8,9,13-trihydroxy-, methyl ester	C_23_H_46_O_5_	402	34.77	-	-	-	0.30
21	9-octadecenoic acid (Z)-, 2-[(trimethylsilyl)oxy]-1-[[(trimethylsilyl) oxy] methyl] ethyl ester	C_27_H_56_O_4_Si_2_	500	38.40	-	-	-	0.28
22	9,12,15-octadecatrienoic acid, 2,3-bis [(trimethylsilyl) oxy] propyl ester, (*Z*,*Z*,*Z*)	C_27_H_52_O_4_Si_2_	496	39.82	-	-	-	1.03
23	Oleic acid, 3-(octadecyloxy) propyl ester	C_39_H_76_O_3_	592	39.89	-	1.73	-	-
24	1,2,3-propanetriyl ester, (*E,E,E*)- 9-Octadecenoic acid	C_57_H_104_O_6_	884	40.64	-	-	-	1.11
25	2-hydroxy-3-[(9*E*)-9-octadecenoyloxy] propyl (9*E*)-9-octadecenoate	C_39_H_72_O_5_	620	41.07	-	-	-	1.48
26	Trilinolein	C_57_H_98_O_6_	878	41.47	-	-	-	3.02
27	Glycidyl oleate	C_21_H_38_O_3_	338	41.87	-	-	-	0.27
					**10.25**	**1.73**	**-**	**14.01**
C. Monoterpenoids								
28	*P*-Menth-8-en-1-ol	C_10_H_18_O	154	9.10	-	-	-	2.79
					**-**	**-**	**-**	**2.79**
D. Sesquiterpenoids								
29	Caryophyllene oxide	C_15_H_24_O	220	6.20	-	-	-	0.63
30	(-)-Spathulenol	C_15_H_24_O	220	18.83	-	-	-	1.75
31	Chamazulene	C_14_H_16_	184	32.78	-	1.27	-	-
					**-**	**1.27**	**-**	**2.38**
E. Diterpenoids								
32	19-Norkaur-16-ene, (4*β*)	C_19_H_30_	258	24.52	-	-	1.16	-
33	Isochiapin B	C_19_H_22_O_6_	346	29.63	-	2.89	-	-
					**-**	**2.89**	**1.16**	**-**
F. Triterpenoids								
34	Lupeol	C_30_H_50_O	426	38.18	-	-	-	3.00
					**-**	**-**	**-**	**3.00**
G. Non-terpenoids hydrocarbons								
35	*Z,Z,Z*-4,6,9-Nonadecatriene	C_19_H_34_	262	6.26	-			0.87
36	3,4-Dihydro-2H-1,5-(3 "-t-butyl) benzodioxepine	C_13_H_18_O_2_	206	17.30	1.45	-	-	-
37	(1-Methylnonyl)- benzene	C_16_H_26_	218	19.29	0.75	-	-	-
38	(1-Pentylhexyl)- benzene	C_17_H_28_	232	19.94	0.90	-	1.32	-
39	(1-Butylheptyl)- benzene	C_17_H_28_	232	20.02	2.51	1.38	3.11	-
40	(1-Propyloctyl)- benzene	C_17_H_28_	232	20.26	1.87	1.18	2.5	-
41	(1-Ethylnonyl)- benzene	C_17_H_28_	232	20.73	2.02	1.25	2.44	-
42	(1-Methyldecyl)-benzene	C_17_H_28_	232	21.59	3.02	2.87	4.35	-
43	(1-Pentylheptyl)-benzene	C_18_H_30_	246	22.11	2.96	2.65	4.24	-
44	(1-Butyloctyl)- benzene	C_18_H_30_	246	22.21	3.35	2.79	4.32	-
45	2H-Pyran, 2-(7-heptadecynyloxy) tetrahydro	C_22_H_40_O_2_	336	22.24	-	-	-	0.48
46	(1-Propylnonyl)—benzene	C_18_H_30_	246	22.48	2.63	2.30	3.40	-
47	(1-Ethyldecyl)- benzene	C_18_H_30_	246	22.95	2.35	2.36	3.24	-
48	(1-Methylundecyl)- benzene	C_18_H_30_	246	23.79	3.58	3.57	6.06	-
49	(1-Pentyloctyl)- benzene	C_19_H_32_	260	24.20	3.43	3.77	5.04	-
50	(1-Butylnonyl)- benzene	C_19_H_32_	260	24.33	2.18	2.19	3.55	-
51	(1-Propyldecyl)- benzene	C_19_H_32_	260	24.60	1.71	2.36	2.65	-
52	(1-Ethylundecyl)- benzene	C_19_H_32_	260	25.08	1.5	2.53	2.4	-
53	(1-Methyldodecyl)- benzene	C_19_H_32_	260	25.89	1.97	2.74	2.88	-
54	2,4-Bis(1-methyl-1-phenylethyl) phenol	C_24_H_26_O	330	36.01	7.26	-	-	-
55	17-Pentatriacontene	C_35_H_70_	490	39.37	-	-	3.00	-
56	Dotriacontane	C_32_H_66_	450	39.42	-	3.26	-	-
57	Tricyclo[20.8.0.0(7,16)]triacontane, 1(22),7(16)-di-epoxy	C_30_H_52_O_2_	444	40.40	-	-	-	4.29
					**45.44**	**37.20**	**54.50**	**5.64**
H. Steroids								
58	14-*β*-H-pregnane	C_21_H_36_	288	29.10	-	2.21	1.47	-
59	Docosahexaenoic acid, 1,2,3-propanetriyl ester	C_69_H_98_O_6_	1022	35.67	-	-	-	0.78
60	Pseduosarsasapogenin-5, 20-diene	C_27_H_42_O_3_	414	36.13	-	-	-	0.86
61	4*β*-Methylandrostane2,3-diol-1,17-dione	C_20_H_30_O_4_	334	36.38	-	-	-	1.61
62	Ethyl iso-allocholate	C_26_H_44_O_5_	436	41.47	-	-	1.79	-
					**-**	**2.21**	**3.26**	**3.25**
I. Alcohol (Alc.)								
63	2-Methyl-*E*,*E*-3,13-octadecadien-1-ol	C_19_H_36_O	280	5.30	-	-	-	0.38
64	1-Hexadecanol, 2-methyl	C_17_H_36_O	797	23.62	-	1.15	-	-
65	*E,E,Z*-1,3,12-Nonadecatriene-5,14-diol	C_19_H_34_O_2_	294	29.40	0.77	-	-	-
66	12-Methyl-*E,E*-2,13-octadecadien-1-ol	C_19_H_36_O	280	30.96	2.51	-	-	-
67	1-Heptatriacotanol	C_37_H_76_O	536	42.83	-	-	-	16.47
					**3.28**	**1.15**	**-**	**16.85**
J. Ketone (Ket.)								
68	7, 9-Ditert-butyl-1-oxaspiro [4.5] deca-6, 9-diene-2, 8-dione	C_17_H_24_O_3_	279	25.78	1.30	-	-	-
					**1.30**	**-**	**-**	**-**
I. Others								
69	2-(Acetyloxy)-1-(hydroxymethyl)ethyl acetate	C_7_H_12_O_5_	176	13.26	-	-	-	0.62
70	7-Heptadecene, 1-chloro-	C_17_H_33_CL	272	23.62	-	-	1.34	-
71	1-Chlorooctadecane	C_18_H_37_Cl	288	25.81	-	2.03	-	-
72	Tert-hexadecanethiol	C_16_H_34_S	258	25.81	-	-	1.62	-
73	9-Oximino-2,7-diethoxyfluorene	C_17_H_17_NO_3_	283	25.96	-	-	-	0.45
					**-**	**2.03**	**2.96**	**1.07**
Total identification					**74.70%**	**50.46%**	**63.45%**	**52.79%**

In where: *M.F* Molecular formula, *M.W.T* Molecular weight, *R.T* Retention time

### Antimicrobial Activity of *C. alexanderina* essential oil extracts and determination of MIC

Antimicrobial activity has been only recorded for inhibition zones with a diameter equal to or more than 12 mm. Roots and stems essential oil extracts demonstrated selective activity against *P. aeruginosa*, while leaves essential oil extract displayed activity against both *S. enterica* and *E. coli* at a concentration of 1 mg/mL. On the contrary, the flowers essential oil extract had no antimicrobial activity against any of the tested strains, and none of the tested essential oil extracts demonstrated efficacy against *C. albicans*. The stems essential oil extract displayed an MIC value of 1 mg/mL against *P. aeruginosa*, whereas leaves essential oil extract showed the same MIC value against both *E*. *coli* and *S. enterica*. However, the MIC value of roots essential oil extract could not be determined up to a dosage of 0.125 mg/mL, indicating that its MIC value was more than 0.125 mg/mL.

### Antioxidant Activity of *C. alexanderina* essential oil extracts

#### DPPH radical-scavenging activity

**Fig. 2:**
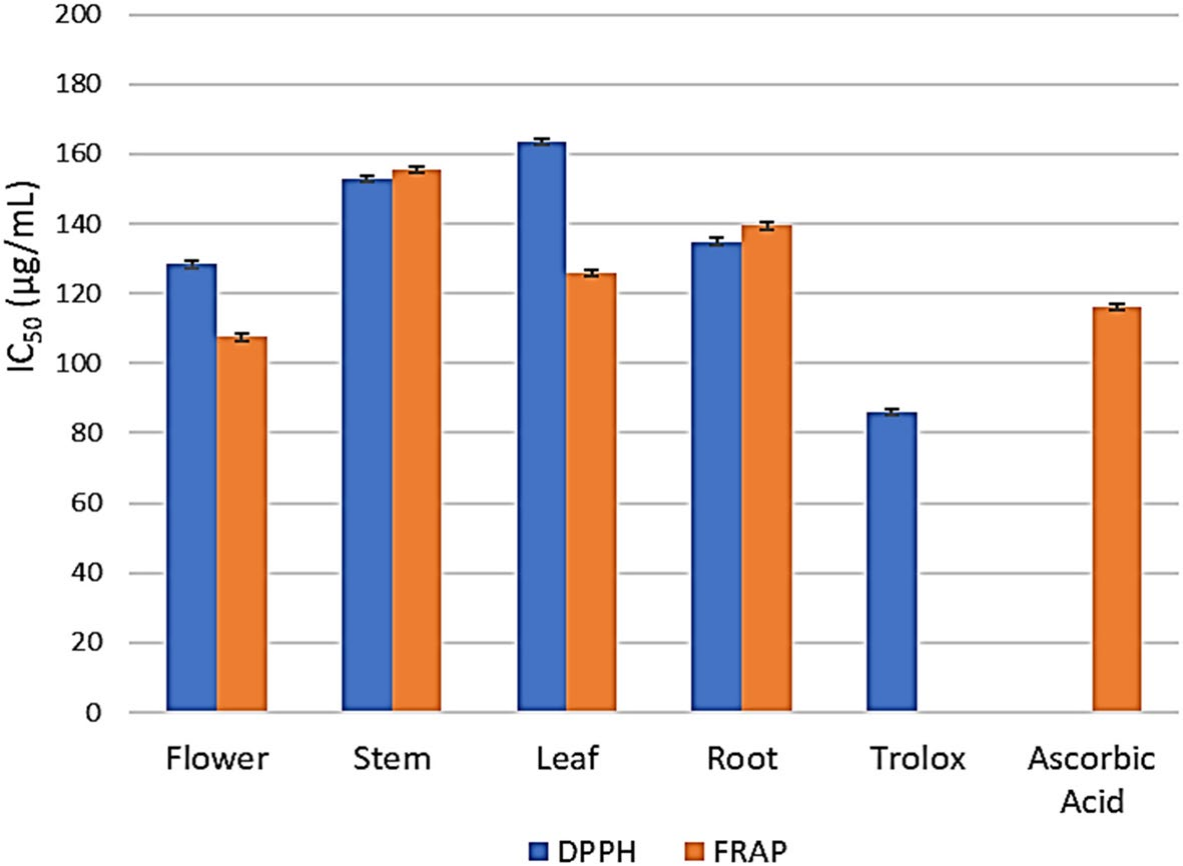
Antioxidant activity of essential oil extracts from different parts of *C. alexanderina* using DPPH and FRAP assays

DPPH reaction was used in the current study to evaluate the overall antioxidant capacity as the cumulative ability of the chemicals present in the sample to scavenge free radicals. The four essential oil extracts generally displayed notable antioxidant capacity as compared to Trolox (IC_50_ = 85.96 ± 3.2 µg/mL) (Table 2S). We were able to order the essential oil extracts of different plant parts in decreasing order of antioxidant activity as follows: the productive part (flowers 128.3 ± 4.8 µg/mL) came first, followed by roots (134.9 ± 5 µg/mL), stems (152.8 ± 5.7 µg/mL), and leaves (163.6 ± 6.1 µg/mL) to convert the radical DPPH into the yellow diphenyl picrylhydrazine (Fig. 2).

#### Ferric Reducing Antioxidant Power (FRAP) Assay

FRAP assay is the only assay that directly measures antioxidants or reductants in a sample. It measures the reducing ability of antioxidant that react with ferric tripyridyltriazine (Fe^3+^ TPTZ) complex and produce a colored ferrous tripyridyltriazine (Fe^2+^–TPTZ). The study showed that the reducing ability of the tested essential oil extracts was in the order of flowers > leaves > roots > stems (107.50 ± 3.64, 125.80 ± 4.2, 139.4 ± 5.27, and 155.59 ± 5.27 ug/mL; respectively) compared to ascorbic acid (116.08 ± 3.93 ug/mL) (Fig. 2, Table 2S).

### *α*-Glucosidase inhibitory activity of *C. alexanderina* essential oil extracts

**Fig. 3:**
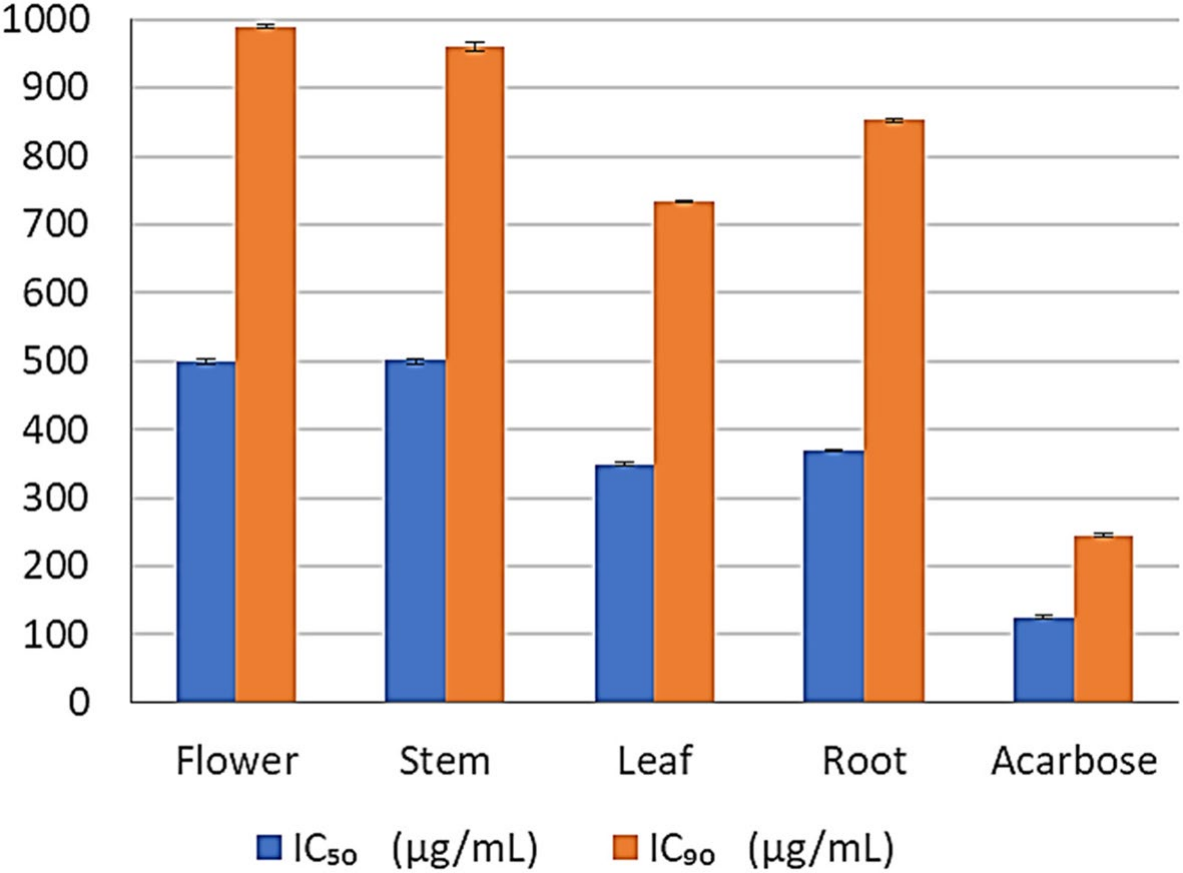
*α*-Glucosidase inhibition activity of essential oil extracts from different parts of *C. alexanderina*

The *α*-Glucosidase inhibitory activity of the four essential oil extracts have been investigated and results are displayed in Fig. 3 as the half-maximal concentration values (IC_50_ and IC_90_ µg/mL). The IC_50_ values ranged from 349 to 501 µg/mL where IC_90_ were between 733.8 to 990 µg/mL. Acarbose was used as a positive control with IC_50_ and IC_90_ of 125 and 245 µg/mL; respectively (Table 3S).

### Molecular docking studies

The metabolites detected in roots and leaves essential oil extracts were docked into *α*-glucosidase enzyme sites using PDB entry 5NN8. Four identified metabolites in roots essential oil extract unveiled binding scores higher than those for the co-crystallized ligands: acarbose in the active site (-7.2 kcal/mol) and acarbose-derived trisaccharides (-7.6 kcal/mol) in the secondary site. On the other hand, the only diterpene (19-norkaur-16-ene) and the two steroids (4*β*-pregnane and ethyl iso-allocholate) that have been detected in leaves essential oil extract were docked well into the secondary site of *α*-glucosidase and unveiled binding affinities (-7.2, -7.3, and -7.0 kcal/mol; respectively) comparable to that of the co-crystallized ligand (-7.6 kcal/mol) (Table 4S).

## Discussion

The phytochemical composition of the essential oil extracts from four parts of *C. alexanderina* (flowers, stems, leaves, and roots) was examined by adopting GC–MS analysis. The results (Table 1 and Fig. 1) revealed various classes of metabolites with the prevalence of non-terpenoid hydrocarbons in flower, stems, and leaves essential oil extracts (45.44%, 37.20%, and 54.50%; respectively), while alcohols were the most predominant class in roots essential oil extract (16.85%). The most prevailing compound detected in flower essential oil extract among twenty-seven identified compounds was hexadecenoic acid (palmitic acid). Palmitic acid is a saturated fatty acid that has been reported to have antimicrobial, anti-inflammatory, antioxidant, and anticancer potentials [28–30]. Another prevailing compound in flower essential oil extract was ethyl (9*Z*,12*Z*)-9,12-octadecadienoate (ethyl linoleate) which has been stated to reduce serum glucose levels in alloxan-induced diabetic rats [31]. The most predominant compounds in stems (22 detected compounds) and leaves essential oil extracts were the non-terpenoid hydrocarbons (1-pentyloctyl)- benzene and (1-methylundecyl)- benzene. On the other hand, among 42 identified constituents in roots essential oil extract, 1-heptatriacotanol (16.47%) was the most prevailing compound that has been reported previously to have a beneficial role as anti-hypercholesterolemia [32], followed by tricyclo[20.8.0.0(7,16)]triacontane, 1(22),7(16)-di-epoxy (4.29%) and oleic acid (3.58%) that has been reported to exert a protective action in breast cancer and anti-inflammatory potential in autoimmune diseases [33].

Reviewing the literature, there is no comparative study regarding the chemical composition of essential oil extracts from *C. alexanderina,* however, Reda et al. reported the results of GC–MS analysis of essential oils from four species of *Centaurea* including *C. alexanderina* where the essential oils were prepared by the conventional hydro-distillation method [11].

From a qualitative perspective, it is noteworthy to highlight that monoterpenoids and triterpenoids were detected only in roots essential oil extract, while diterpenoids were present solely in leaves and stems essential oil extracts. Fatty acid esters were absent in leaves essential oil extract, and ketones appeared only in flower essential oil extract. On the other hand, sesquiterpenoids were detectable only in stems and roots essential oil extracts, while the presence of alcohol was limited to flowers and roots essential oil extracts where alcohols constituted the most abundant chemical class in root essential oil extract.

Previous work has reported the antimicrobial potential of several *Centaurea* species. *C. tweediei* has been reported to have a remarkable activity against methicillin-resistant *Staphylococcus aureus* [34], while *C. cyanus* L., *C. jacea* L., and *C. scabiosa* L manifested low antimicrobial activities against *Agrobacterium tumefaciens*, *Erwinia amylovora*, *Erwinia carotovora*, *Pantoea agglomerans*, *Pseaudomonas syringae*, and *Xanthomonas arboricola* [35]. *C. odyssei* and *C. kurdica* from Turkey depicted significant activity against *Staphylococcus aureus, Micrococcus luteus, Bacillus cereus, Salmonella typhimurium, Pseudomonas aeruginosa,* and *Klebsiella pneumoniae* [36].

Antimicrobial activity of the plant essential oil extracts has been explored. The bacterial strains were selected to be representative of both common Gram- negative pathogens such as *E. Coli* (major cause of various GIT and urinary tract infections and neonatal meningitis), *S. enterica* (the main pathogen causing typhoid fever and parathyroid fever), and *P. aeruginosa* (one of the most common opportunistic pathogens worldwide causing wide range of infections) as well as the Gram-positive pathogens such as the vancomycin resistant *E. faecalis* (causing wide range of human infections and one of the ESKAPE pathogens group in addition to its high microbial resistance to most available antibiotics including the last choice drugs of Gram-positive bacteria) and *L. monocytogenes* (one of the most common food pathogens). Based upon the above recorded results, the main components detected during the chemical profiling may play a role either solely or synergistically in the antimicrobial activity of leaves, stems, and roots essential oil extracts against *E.coli, S. enterica and P. aeruginosa,* For instance; sesquiterpenes as spathulenol [11] and chamazulene [37], non-terpenoid hydrocarbons as 17-pentatriacontene [38], and steroids as ethyl iso-allocholate [39] have been reported to have activity against both *P. aeruginosa* and *E. coli*. It’s noteworthy that leaf methanolic extract of *C. alexanderina* was previously reported to be active against *P. aeruginosa*, while its essential oil prepared by hydro-distillation showed activity against both *S. aureus* and *E. coli* [9, 11].

In the current study, *C. alexanderina* has been investigated for the first time for its antioxidant potential. The four essential oil extracts of the plant were assayed for their antioxidant capacity using two different approaches, (DPPH and FRAP). Investigation of antioxidant capacity using DPPH assay that relies on the principle of measuring the scavenging ability of DPPH free radical revealed that the flowers essential oil extract exhibited the highest antioxidant capacity, while the leaves essential oil extract manifested the lowest activity. In FRAP assay, the indicator is the reduction of ferric (in the form of ferric tripyridyltriazine (Fe^3+^‐TPTZ) salt solution) to ferrous ion by the action of the antioxidant under reaction conditions where the color changes from pale yellow to blue and the absorbance at 594 nm increases [40]. Using FRAP assay, results confirmed the most antioxidant capacity for flowers essential oil extract. On the other hand, leaves essential oil extract unveiled more antioxidant capacity than roots and stems. It was not surprising that DPPH and FRAP assays would give different results, this difference is attributed to different reaction mechanisms [41]. Previous reports on other species of *Centaurea* confirmed the antioxidant potential of their extracts and essential oils [42–45].

Diabetes mellitus is a chronic metabolic syndrome that has significant social, health, and economic implications. There are two types of diabetes (1 and 2); Type 2 is more common all over the world [46]. It causes dangerous complications especially on the cardiovascular system. Different pathways are intended for preventing the progression of that disease, one of which is the *α*-glucosidase inhibition. *α*-Glucosidase enzyme is involved in the digestion of carbohydrates, so it can significantly reduce the post-prandial glucose level. Clinically used compounds for treatment of type 2 diabetes mellitus showed different side effects as swelling, abdominal distraction, diarrhea, and meteorism, it also needs a great attention [47]. So, there is a demand to search for the herbal remedies for treatment of type 2 diabetes mellitus. In Egypt, people have been used *Centaurea* species as anti-hyperglycemic drug [48]. Herein, the highest *α*-glucosidase inhibitory activity was observed for leaves and roots essential oil extracts with IC_50_ of 349 ± 3.7 and 369 ± 5.16 µg/mL, respectively. At the same time, stems essential oil extract exhibited the lowest activity with IC_50_ value of 501 ± 4.8 µg/mL. *α*-Glucosidase inhibitory activity of roots essential oil extract may be due to the fatty acid and fatty acid esters content particularly; *cis*-5, 8, 11, 14, 17-eicosapentaenoic acid (0.48%) [49]. Lupeol, as well, was reported to inhibit *α*-glucosidase [50], it was detected only in roots sample.

Molecular docking studies have been performed to explore the possible interactions between detected compounds in the most active essential oil extracts and the antidiabetic target, *α*-glucosidase enzyme. On a molecular basis, the crystal structure of recombinant human *α*-glucosidase enzyme (rhGAA) embraces more than one glycosylation site, previous research on complex of the inhibitor acarbose with *α*-glucosidase revealed that acarbose was bound in a catalytic site (the active site) and established interactions with Asp616, Asp282, and Arg600, moreover it was observed that a second acarbose moiety (acarbose-derived trisaccharide) was inserted into another pocket (secondary substrate-binding site) about 25 Å away from the active site and was stabilized via interaction with Cys127, Ala97, Asp91, Trp126, and Pro125 [24]. Herein, we examined the binding affinity of GC–MS identified compounds in leaves and roots essential oil extracts with rhGAA.

**Fig. 4:**
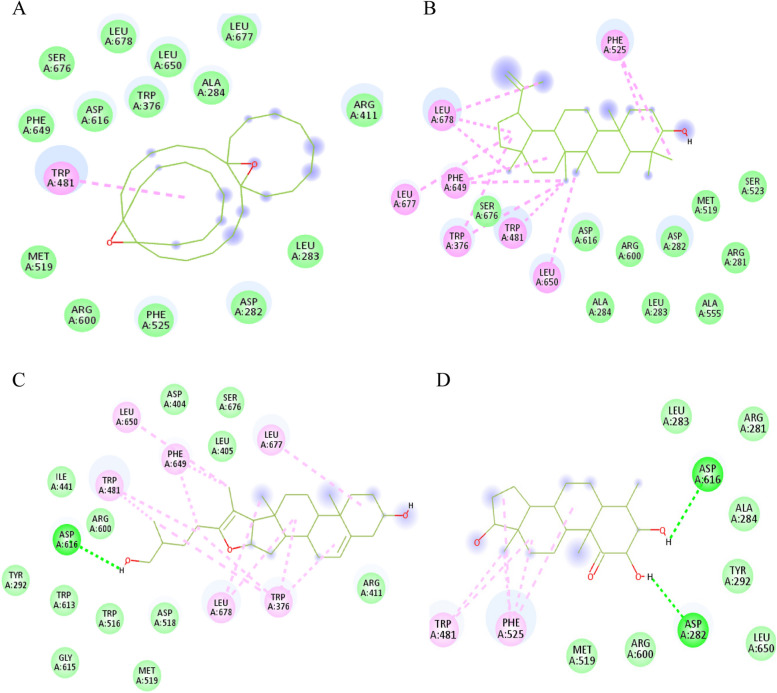
2D interactions of *α*-glucosidase active site residues with tricyclo [20.8.0.0 (7,16)] triacontane,1(22),7(16)-di-epoxy (**A**), lupeol (**B**), pseduosarsasapogenin-5,20-dien (**C**), and 4*β*-methylandrostane 2,3-diol-1,17-dione (**D**) detected in roots essential oil extract of *C. alexanderina*

**Fig. 5:**
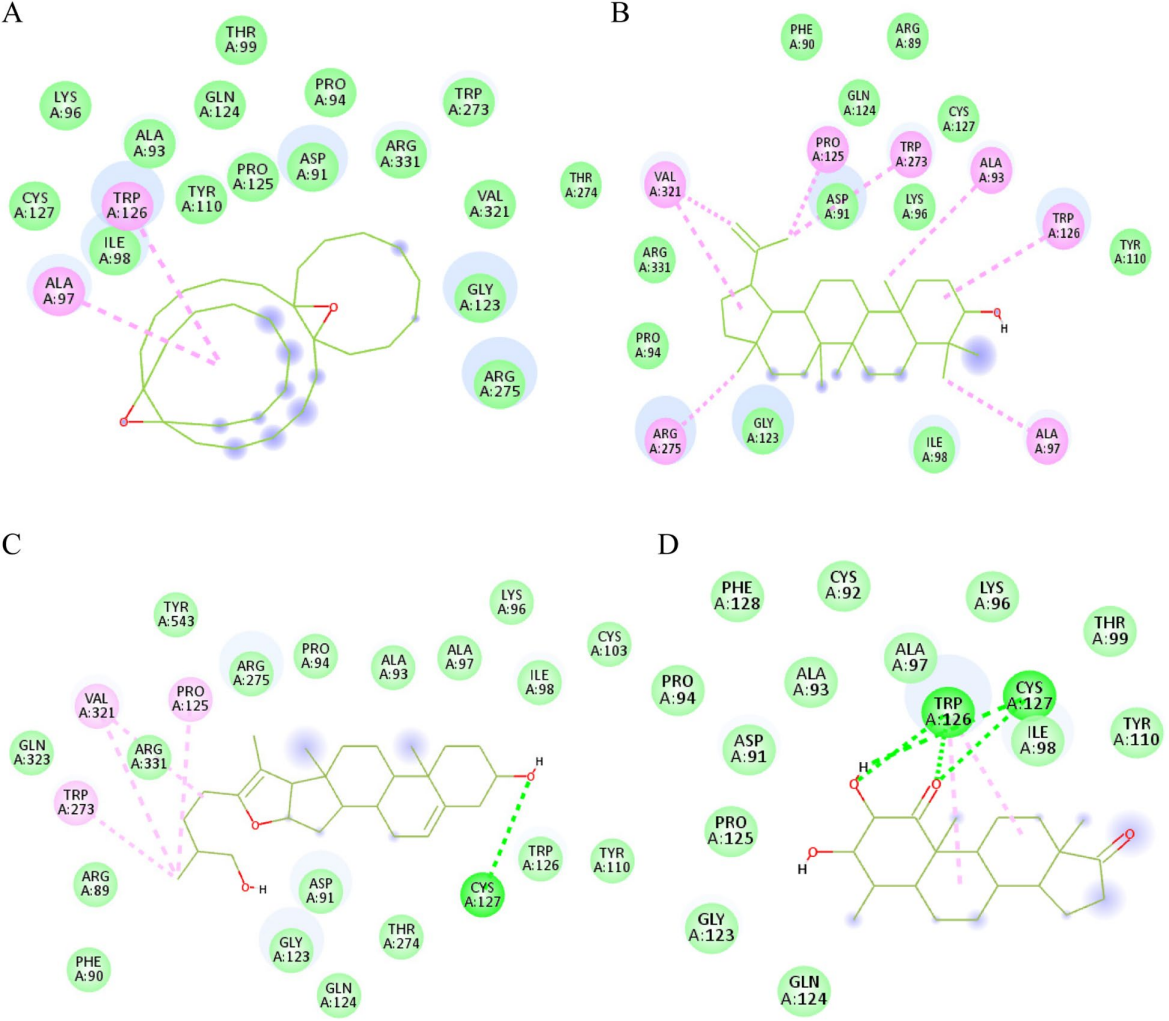
2D interactions of *α*-glucosidase secondary substrate-binding site residues with tricyclo [20.8.0.0 (7,16)] triacontane,1(22),7(16)-di-epoxy (**A**), lupeol (**B**), pseduosarsasapogenin-5,20-dien (**C**), and 4*β*-methylandrostane 2,3-diol-1,17-dione (**D**) detected in roots essential oil extract of *C. alexanderina*

Among the metabolites that detected solely in root essential oil extract, 4 metabolites; namely tricyclo[20.8.0.0(7,16)]triacontane, lupeol, 1(22),7(16)-di-epoxy pseduosarsasapogenin-5,20-diene, and 4*β*-methylandrostane2,3-diol-1,17-dione unveiled high binding affinities to both the active site and secondary site of rhGAA, On the other hand, ethyl iso-allocholate and 19-norkaur-16-ene, (4*β*)- were detected in leaves essential oil extract only and exhibited high binding affinities to both sites of the enzyme. This may explain the high inhibitory effects of both extracts against *α*-glucosidase enzyme in vitro to be due to synergistic action of the mentioned metabolites in addition to the remaining detected compounds that revealed moderate binding affinities to rhGAA. The hydrocarbon, tricyclo [20.8.0.0 (7,16)] triacontane,1(22),7(16)-di-epoxy lodged itself in *α*-glucosidase active site with binding affinity of -7.9 kcal/mol, it exhibited π-alkyl interaction with Trp481 and its pose was stabilized via fourteen hydrophobic interactions (Fig.  4A), it fitted as well in the secondary site (-8.3 kcal/mol) via two π-alkyl interactions with Ala97 and Trp126, in addition to fifteen hydrophobic contacts (Fig.  5A). The triterpene: lupeol was embedded in the hydrophobic cavity, which is formed by Trp376, Trp481, Phe525, Phe649, Leu650, Leu677, and Leu678 in the active site (-7.4 kcal/mol) and twelve hydrophobic interactions were detected (Fig.  4B), moreover, lupeol well accommodated into the secondary site and established eight alkyl interactions with Ala93, Ala97, Pro125, Trp126, Trp273, Arg275, and Val321 (Fig. 5B). It is worthy to note that lupeol antidiabetic activity on diabetic rats and its *α-*glucosidase inhibition have been previously outlined [51, 52]. The sapogenin; pseduosarsasapogenin-5,20-dien depicted a hydrogen bond with the catalytic Asp616 and ten alkyl interactions with Trp376, Trp481, Phe649, Leu650, Leu677, and Leu678 in the active site (Fig. 4C) and bound as well in the secondary site via a hydrogen bond to Cys127, four alkyl interactions with Pro125, Trp273, and Val321 and eighteen hydrophobic contacts (Fig. 5C). Finally, the steroid; 4*β*-methylandrostane 2,3-diol-1,17-dione was inserted into the active site and exhibited two hydrogen bonds formed between its hydroxyl groups at C-2 and C-3 and Asp282 and Asp616, respectively, in addition to five π-alkyl interactions between rings B, C, and D of its androstane nucleus and the amino acids; Trp481 and Phe525, moreover, seven hydrophobic contacts with Arg281, Leu283, Ala284, Tyr292, Met519, Arg600, and Leu650 were observed (Fig. 4D), while in the secondary site, the hydroxyl group at C-2 formed two hydrogen bonds with Trp126 and Cys127 while carbonyl group at C-1 exhibited two hydrogen bonds with the same residues in addition to two π-alkyl interactions with Trp126 and thirteen hydrophobic contacts (Fig. 5D).

**Fig. 6:**
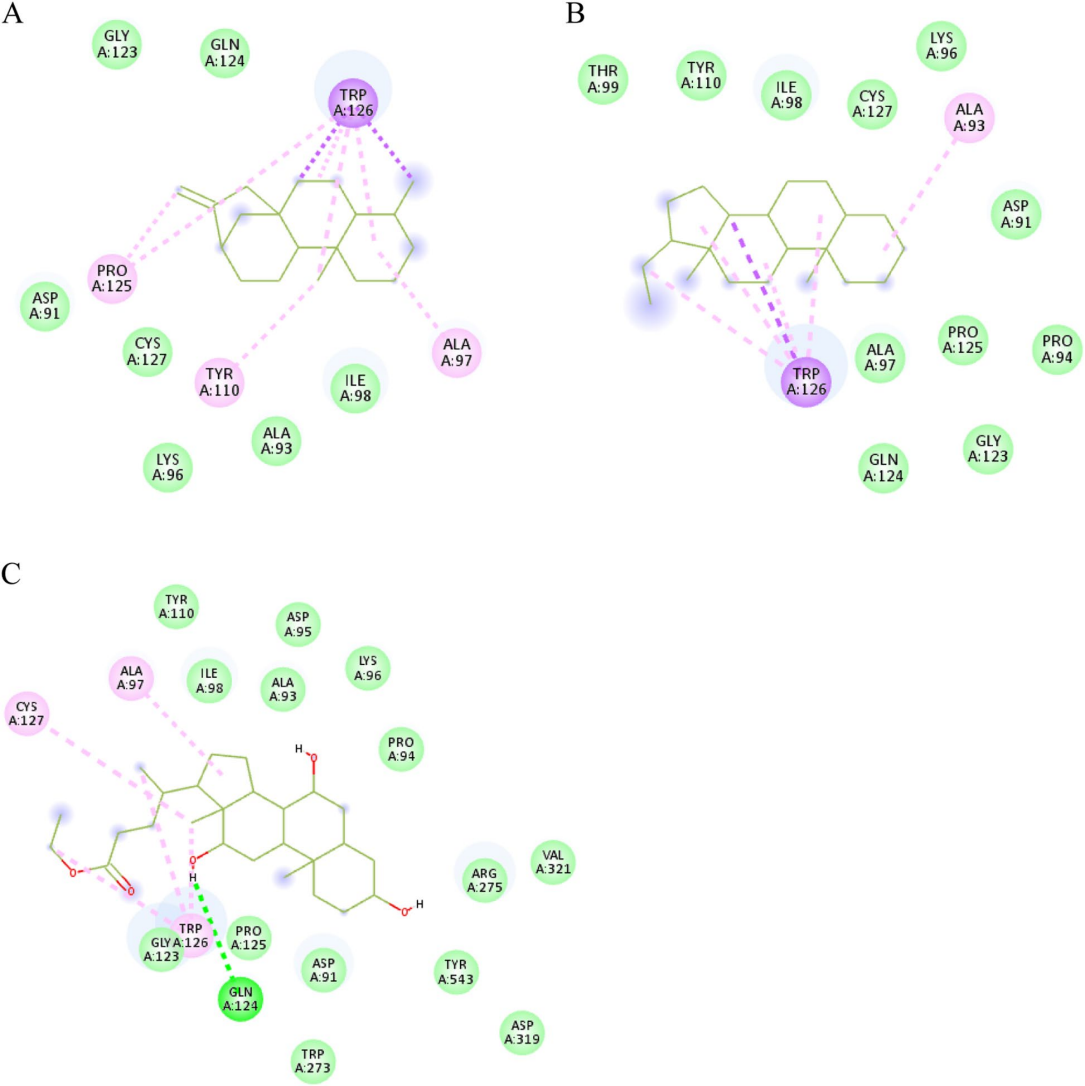
2D interactions of *α*-glucosidase secondary substrate-binding site residues with 19-norkaur-16-ene (**A**), 4*β*-pregnane (**B**), and ethyl iso-allocholate (**C**) detected in leaves essential oil extract of *C. alexanderina*

19-norkaur-16-ene, 4*β*-pregnane and ethyl iso-allocholate; in leaves essential oil extract; exhibited π-alkyl interactions with Trp126, furthermore, 19-norkaur-16-ene was more stabilized via two π-σ interactions with Trp126, in addition to four alkyl interactions with Ala97, Tyr110, and Pro125 and seven van der Waals contacts (Fig. 6A). Similarly, the steroid 4*β*-pregnane which was inserted into the secondary site and formed one π-σ interaction with Trp126, one alkyl interaction with Ala93, and van der Waals interactions with eleven residues (Fig. 6B), while the steroid ethyl iso-allocholate depicted one hydrogen bonding with Gln124 in addition to two alkyl interactions with Ala97 and Cys127 (Fig. 6C). Ethyl iso-allocholate has been previously isolated from *Trigonella foenum graecum* and reported to have cytotoxic activity [53] but no previous reports on its *α-*glucosidase inhibition activity. The overall docking findings suggested leaves and roots essential oil extracts as sources for *α*-glucosidase inhibitors that could be beneficial for development of antidiabetic drugs.

## Conclusions

The essential oil extracted from different fresh parts of *C. alexanderina* have been studied for the first time to analyze their chemical components, as well as their antimicrobial, antioxidant, and anti-hyperglycemic effects. Experimental findings revealed significant quantitative and qualitative variations in the composition of the four essential oil extracts. Notably, non-terpenoid hydrocarbons were the dominant compounds in the essential oil extracts derived from flowers, stems, and leaves, whereas alcohols were the predominant class in roots essential oil extracts. Roots and stems essential oil extracts demonstrated selective activity against *P. aeruginosa*, while leaves essential oil extract displayed activity against both* S. enterica* and *E. coli.* The antioxidant properties of the essential oil extracts were evaluated using DPPH and FRAP assays, where the flower essential oil extract exhibited the highest antioxidant potential in both tests. Leaves and roots essential oil extracts revealed more *α*-glucosidase inhibition compared to flowers and stems, an effect that has been supported by molecular docking results which revealed high binding affinities of GC–MS identified compounds in leaves and roots essential oil extracts with rhGAA. These findings suggested *C. alexanderina* flower essential oil extract for possible use as natural antioxidant. Furthermore, roots and leaves essential oil extracts could be possible inhibitors for *α*-glucosidase enzyme. Further studies are recommended for more investigation.

## Supplementary Information

Supplementary Material 1

## Data Availability

All data generated or analysed during this study are included in this published article.
