# Supplementary material for: A comparative study of the essential oil extracts of Centaurea alexanderina different parts: GC-MS analysis, antimicrobial, antioxidant, and anti-hyperglycemic activities
Source: BMC Complement Med Ther. 2025 Mar 5;25:90. doi: 10.1186/s12906-025-04805-1 (PMC11883959; doi:10.1186/s12906-025-04805-1)
Supplement: Supplementary file 1 — Supplementary Material 1 [file 12906_2025_4805_MOESM1_ESM.docx]

**A comparative study of the essential oil extracts of *Centaurea alexanderina* different parts: GC-MS analysis, antimicrobial, antioxidant and anti-hyperglycemic activities.**

Hossam A. Abdallah^1#^, Naglaa Afifi^1#^, Enas I. A. Mohamed^1^, Mohamed Sebak^2^, Rabab Mohammed^1*^, Mohamed A. Zaki^1^

^1^ Department of Pharmacognosy, Faculty of Pharmacy, Beni-Suef University, Beni-Suef 62514, Egypt

^2^ Microbiology and Immunology Department, Faculty of Pharmacy, Beni-Suef University, 62514, Egypt.

^#^ These authors contributed equally to this work.

^*^ **Corresponding author:** Rabab Mohammed

E.mail: [rababmohammed@pharm.bsu.edu.eg](mailto:rababmohammed@pharm.bsu.edu.eg) & rmwork06@yahoo.com

Orcid no: <https://orcid.org/0000-0001-9683-4250>

| **A** | 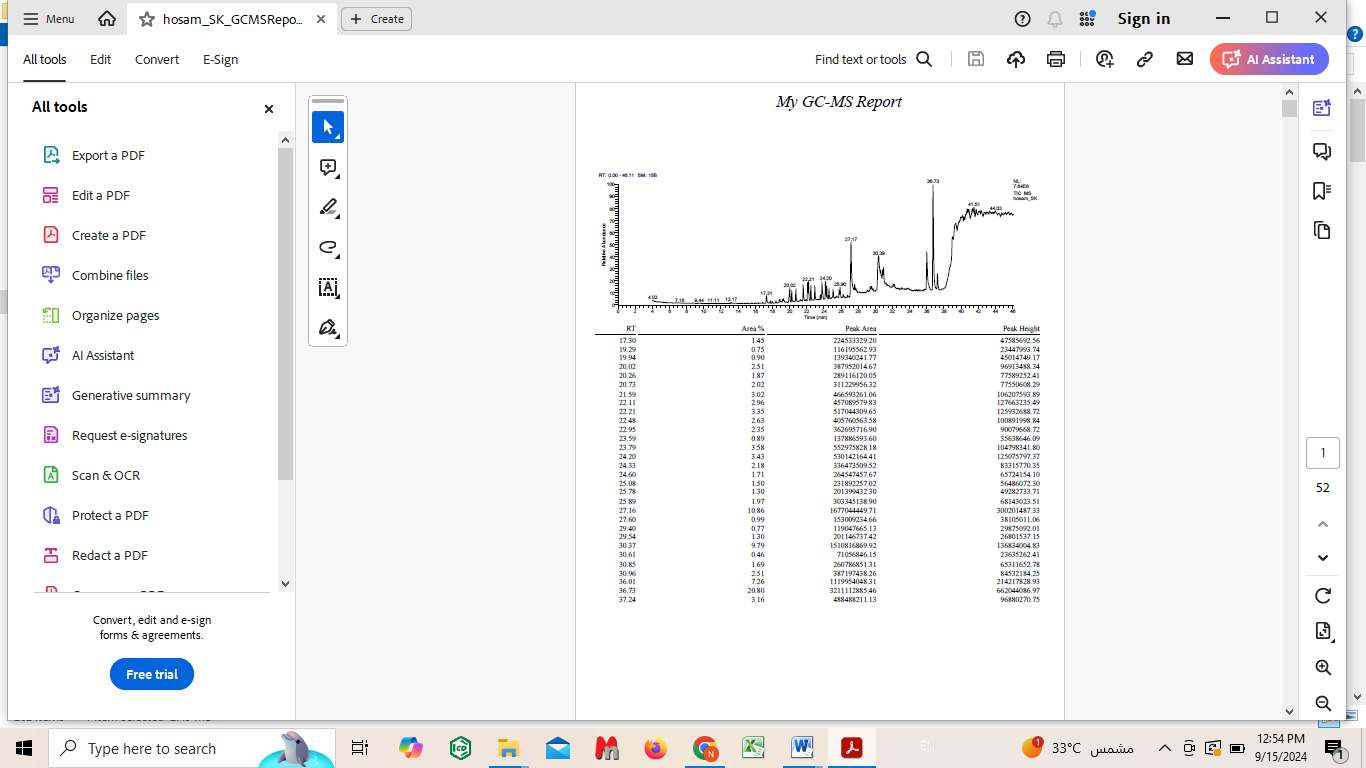  **54**  **17**  **3** |
| --- | --- |
| **B** | 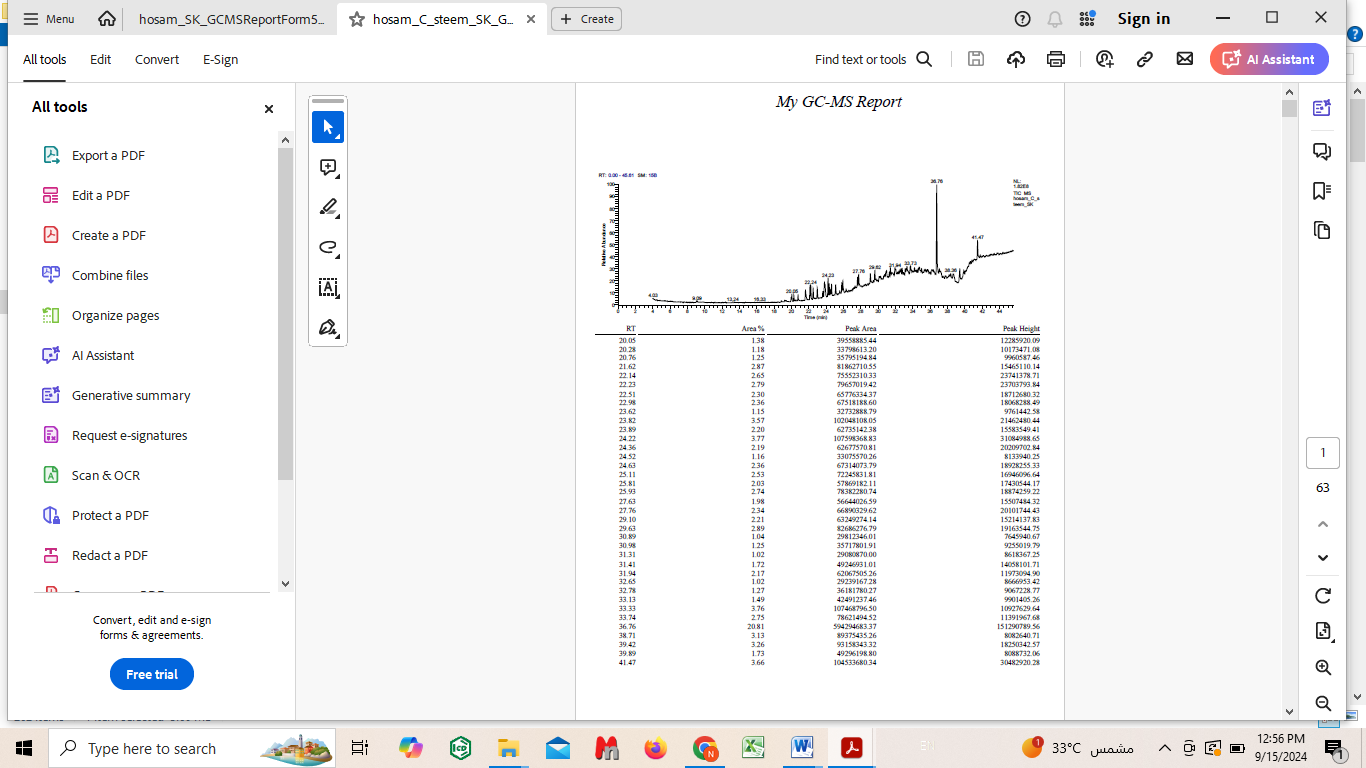  **49**  **48** |
| **C** | 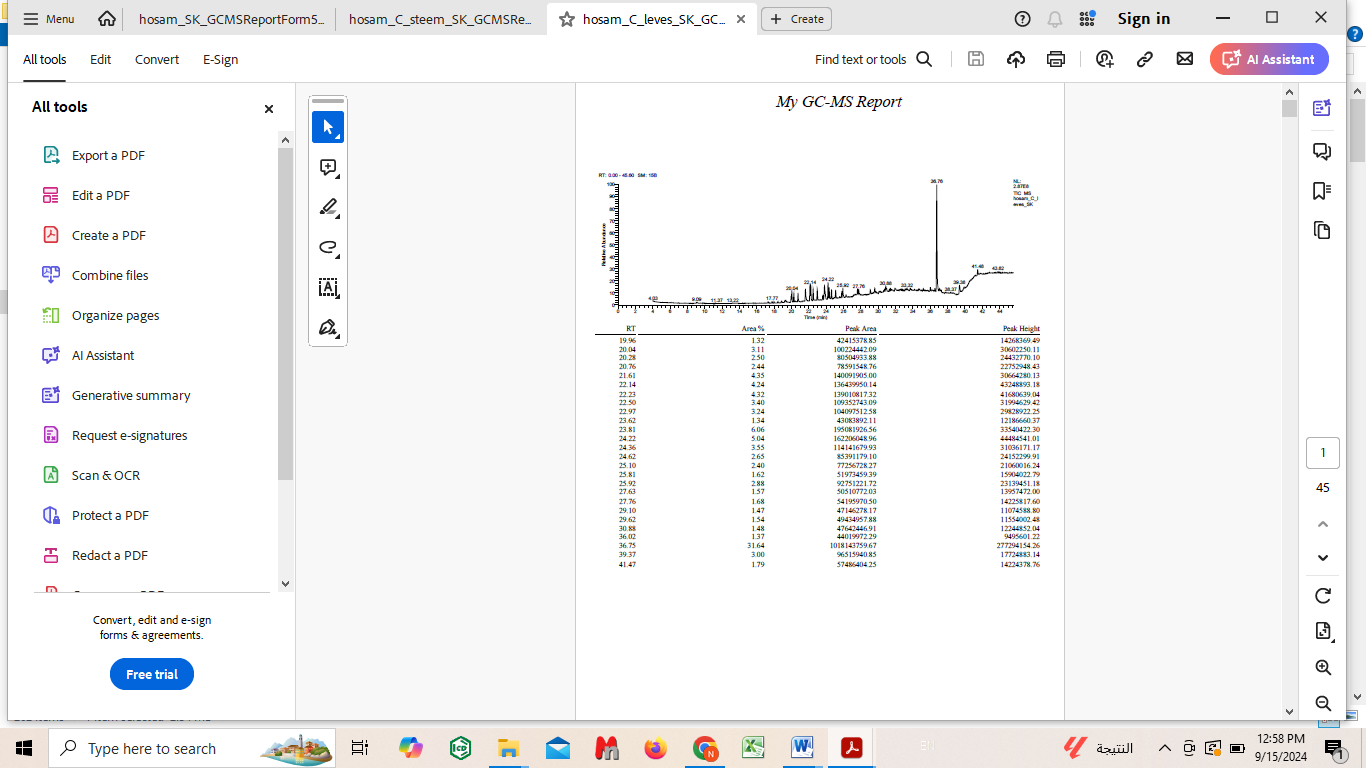  **67**  **49**  **48** |
| **D** | 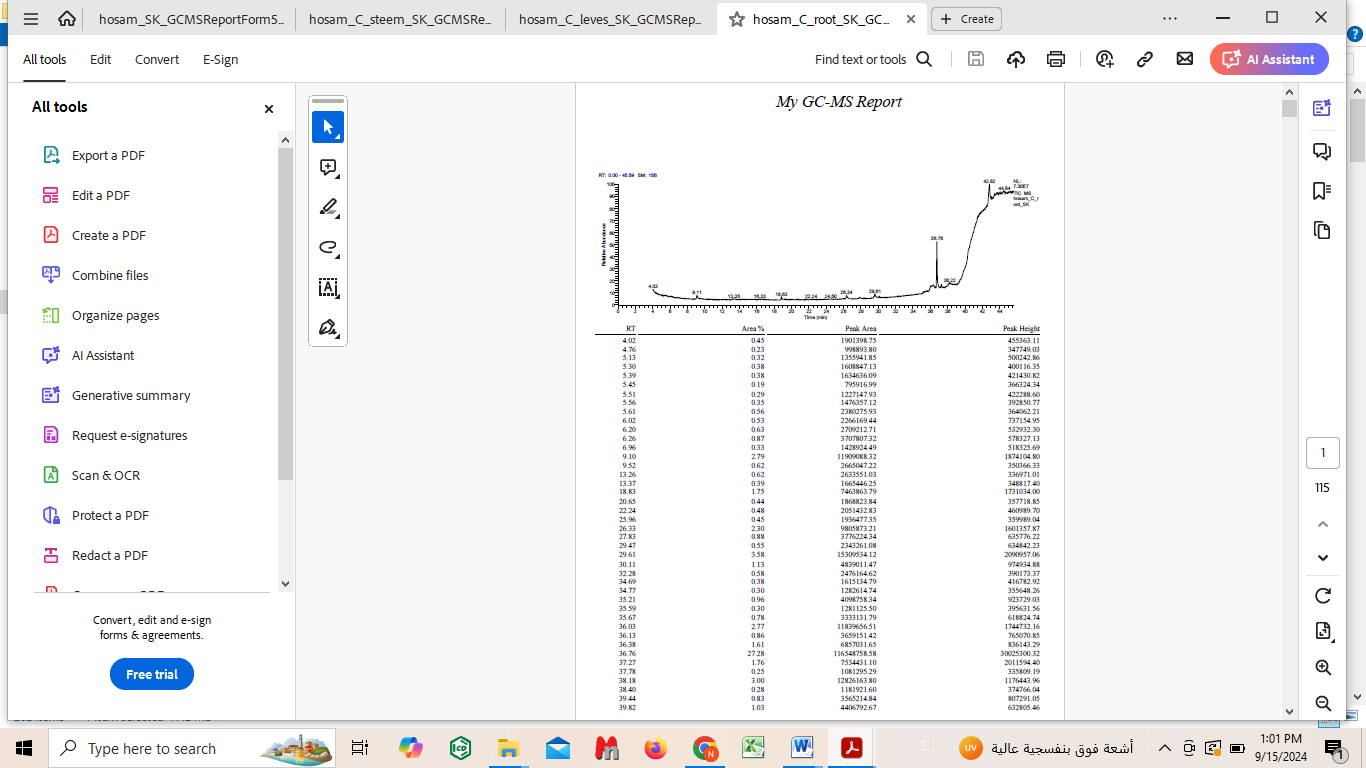 |

**Figure 1S. GC-MS Chromatograms of essential oil extracts from different parts of *C. alexanderina*: (A): Flowers, (B): Stems, (C): Leaves, (D): Roots**

**Table 1S: Result of the antimicrobial activity of *C. alexanderina* essential oil extracts.**

| **Extract** | ***Listeria monocytogenes*** | ***Enterococcus faecalis*** | ***Escherichia. coli*** | ***Salmonella enterica*** | ***Pseudomonas aeruginosa*** | ***Candida albicans*** |
| --- | --- | --- | --- | --- | --- | --- |
| **Flower** | NA | NA | NA | NA | NA | NA |
| **Stem** | NA | NA | NA | NA | + | NA |
| **Leaf** | NA | NA | + | + | NA | NA |
| **Root** | NA | NA | NA | NA | + | NA |
| **Ciprofloxacin** | + | + | + | + | + | NA |
| **Nystatin** | NA | NA | NA | NA | NA | + |

Antimicrobial activity was only recorded for inhibition zones with a diameter equal to or more than 12 mm. NA = inactive

**Table 2S: Antioxidant activity of *C. alexanderina* essential oil extracts**

| **Extract** | **Antioxidant Capacity (IC_50_, μg/mL)** | |
| --- | --- | --- |
|  | **DPPH** | **FRAP** |
| **Flower** | 128.30 ± 4.80 | 107.50 ± 3.64 |
| **Stem** | 152.80 ± 5.70 | 155.59 ± 5.27 |
| **Leaf** | 163.60 ± 6.10 | 125.80 ± 4.26 |
| **Root** | 134.90 ± 5.00 | 139.40 ± 4.72 |
| **Trolox** | 85.96 ± 3.20 | - |
| **Ascorbic acid** | - | 116.08 ± 3.93 |

**Table 3S: *α*-Glucosidase inhibitory activity of *C. alexanderina* essential oil extracts**

| **Extract** | ***α-*Glucosidase Inhibition (μg/mL)** | |
| --- | --- | --- |
|  | **IC_50_** | **IC_90_** |
| **Flowers** | 499.4 ± 4.6 | 990.0 ± 4.43 |
| **Stems** | 501.0 ± 4.8 | 960.1 ± 5.8 |
| **Leaves** | 349.0 ± 3.7 | 733.8 ± 4.1 |
| **Roots** | 369.0 ± 5.16 | 852.8 ± 3.28 |
| **Acarbose** | 125.0 ± 3 | 245.0 ± 3.7 |

**Table 4S: Binding affinities of detected metabolites in the essential oil extracts of *C. alexanderina* to the active site and secondary site of *α*-glucosidase enzyme.**

| **No.** | **Compound name** | **Molecular formula** | **Binding affinity (kcal/mol)** | |
| --- | --- | --- | --- | --- |
|  |  |  | **Active site** | **Secondary site** |
| 1 | *Cis*-5, 8, 11, 14, 17-Eicosapentaenoic acid | C_20_H_30_O_2_ | -5.9 | -5.9 |
| 4 | Oleic acid | C_18_H_34_O_2_ | -5.3 | -4.9 |
| 6 | *Cis*-13-Eicosenoic acid | C_20_H_38_O_2_ | -5.1 | -5.0 |
| 8 | 2,3-dihydroxypropyl ester, (Z, Z, Z)-9,12,15-octadecatrienoic acid | C_21_H_36_O_4_ | -5.6 | -4.9 |
| 9 | 6,9,12,15-Docosatetraenoic acid methyl ester | C_23_H_38_O_2_ | -5.3 | -4.8 |
| 10 | 10-Heptadecen-8-ynoic acid methyl ester(E) | C_18_H_30_O_2_ | -5.5 | -5.5 |
| 11 | 5,8,11,14-Eicosatetraenoic acid, methyl ester (all-Z) | C_21_H_34_O_2_ | -5.5 | -5.3 |
| 12 | *Cis*-2-phenyl-1, 3-dioxolane-4-methyl octadec-9, 12, 15-trienoate | C_28_H_40_O_4_ | -6.0 | -6.1 |
| 13 | 9-Octadecen-12-ynoic acid, methyl ester | C_19_H_32_O_2_ | -5.5 | -4.7 |
| 14 | Methyl 16-hydroxy-hexadecanoate | C_17_H_34_O_3_ | -5.3 | -4.7 |
| 15 | Hexadecadienoic acid, methyl ester | C_17_H_30_O_2_ | -5.0 | -4.4 |
| 16 | Cyclopropanedodecanoic acid, 2-octyl-, methyl ester | C_24_H_46_O_2_ | -5.1 | -4.8 |
| 19 | Hexadecanoic acid, 2,3-dihydroxypropyl ester | C_19_H_38_O_4_ | -5.0 | -4.8 |
| 20 | Docosanoic acid, 8,9,13-trihydroxy-, methyl ester | C_23_H_46_O_5_ | -5.5 | -5.5 |
| 24 | 1,2,3-propanetriyl ester, (*E,E,E*)- 9-Octadecenoic acid | C_57_H_104_O_6_ | -4.4 | -3.9 |
| 25 | 2-hydroxy-3-[(9 *E*)-9-octadecenoyloxy] propyl(9*E*)-9-octadecenoate | C_39_H_72_O_5_ | -5.8 | -4.5 |
| 27 | Glycidyl oleate | C_21_H_38_O_3_ | -5.6 | -5.1 |
| 28 | *P*-Menth-8-en-1-ol | C_10_H_18_O | -5.5 | -5.2 |
| 29 | Caryophyllene oxide | C_15_H_24_O | -5.5 | -6.0 |
| 30 | (-)-Spathulenol | C_15_H_24_O | -5.5 | -6.3 |
| 32 | 19-norkaur-16-ene, (4*β*)- | C_19_H_30_ | -6.3 | -7.2 |
| 34 | Lupeol | C_30_H_50_O | -7.4 | -7.9 |
| 38 | (1-pentylhexyl)- benzene | C_17_H_28_ | -5.6 | -4.8 |
| 39 | (1-butylheptyl)- benzene | C_17_H_28_ | -6.0 | -5.1 |
| 40 | (1-propyloctyl)- benzene | C_17_H_28_ | -6.7 | -5.8 |
| 41 | (1-ethylnonyl)- benzene | C_17_H_28_ | -5.8 | -6.0 |
| 42 | (1-methyldecyl)-benzene | C_17_H_28_ | -6.1 | -5.2 |
| 43 | (1-pentylheptyl)-benzene | C_18_H_30_ | -6.2 | -4.8 |
| 44 | (1-butyloctyl)- benzene | C_18_H_30_ | -6.6 | -5.9 |
| 45 | 2H-Pyran, 2-(7-heptadecynyloxy) tetrahydro | C_22_H_40_O_2_ | -5.4 | -5.0 |
| 46 | (1-propylnonyl)- benzene | C_18_H_30_ | -6.6 | -4.9 |
| 47 | (1-ethyldecyl)- benzene | C_18_H_30_ | -6.3 | -4.6 |
| 48 | (1-methylundecyl)- benzene | C_18_H_30_ | -6.7 | -5.4 |
| 49 | (1-pentyloctyl)- benzene | C_19_H_32_ | -6.4 | -5.8 |
| 50 | (1-butylnonyl)- benzene | C_19_H_32_ | -6.6 | -5.1 |
| 51 | (1-propyldecyl)- benzene | C_19_H_32_ | -6.8 | -5.7 |
| 52 | (1-ethylundecyl)- benzene | C_19_H_32_ | -6.6 | -5.3 |
| 53 | (1-methyldodecyl)- benzene | C_19_H_32_ | -6.4 | -5.8 |
| 55 | 17-pentatriacontene | C_35_H_70_ | -5.3 | -4.7 |
| 57 | Tricyclo[20.8.0.0(7,16)]triacontane, 1(22),7(16)-di-epoxy | C_30_H_52_O_2_ | -7.9 | -8.3 |
| 58 | 14-*β*-H-pregnane | C_21_H_36_ | -6.8 | -7.3 |
| 59 | Docosahexaenoic acid, 1,2,3-propanetriyl ester | C_69_H_98_O_6_ | -4.4 | -4.5 |
| 60 | Pseduosarsasapogenin-5, 20-diene | C_27_H_42_O_3_ | -7.4 | -7.9 |
| 61 | 4*β*-Methylandrostane2,3-diol-1,17-di one | C_20_H_30_H_4_ | -7.3 | -7.9 |
| 62 | Ethyl iso-allocholate | C_26_H_44_O_5_ | -6.2 | -7.0 |
| 63 | 2-Methyl-E,E-3,13-octadecadien-1-ol | C_19_H_36_O | -5.4 | -4.6 |
| 67 | 1-heptatriacotanol | C_37_H_76_O | -4.9 | -4.5 |
|  | Acarbose | C_25_H_43_NO_18_ | -7.2 | - |
|  | Acarbose derived trisaccharide | C_19_H_33_NO_13_ | - | -7.6 |
